# Hyper-SUMOylation of ERG Is Essential for the Progression of Acute Myeloid Leukemia

**DOI:** 10.3389/fmolb.2021.652284

**Published:** 2021-03-26

**Authors:** Xu Chen, Yuanyuan Qin, Zhenzhen Zhang, Zhengcao Xing, Qiqi Wang, Wenbin Lu, Hong Yuan, Congcong Du, Xinyi Yang, Yajie Shen, Biying Zhao, Huanjie Shao, Xiaotong Wang, Hongmei Wu, Yitao Qi

**Affiliations:** ^1^Key Laboratory of the Ministry of Education for Medicinal Resources and Natural Pharmaceutical Chemistry, National Engineering Laboratory for Resource Developing of Endangered Chinese Crude Drugs in Northwest of China, College of Life Sciences, Shaanxi Normal University, Xi’an, China; ^2^School of Agriculture, Ludong University, Yantai, China

**Keywords:** ERG, SUMO2, SENP2, PML, AML

## Abstract

Leukemia is a malignant disease of hematopoietic tissue characterized by the differentiation arrest and malignant proliferation of immature hematopoietic precursor cells in bone marrow. ERG (ETS-related gene) is an important member of the E26 transformation-specific (ETS) transcription factor family that plays a crucial role in physiological and pathological processes. However, the role of ERG and its modification in leukemia remains underexplored. In the present study, we stably knocked down or overexpressed ERG in leukemia cells and observed that ERG significantly promotes the proliferation and inhibits the differentiation of AML (acute myeloid leukemia) cells. Further experiments showed that ERG was primarily modified by SUMO2, which was deconjugated by SENP2. PML promotes the SUMOylation of ERG, enhancing its stability. Arsenic trioxide decreased the expression level of ERG, further promoting cell differentiation. Furthermore, the mutation of SUMO sites in ERG inhibited its ability to promote the proliferation and inhibit the differentiation of leukemia cells. Our results demonstrated the crucial role of ERG SUMOylation in the development of AML, providing powerful targeted therapeutic strategies for the clinical treatment of AML.

## Introduction

Leukemia is a severe clonal disease of the hematopoietic system characterized by the differentiation arrest and malignant proliferation of immature hematopoietic precursor cells in bone marrow (Juliusson and Hough, 2016). Leukemia is caused by genetic and epigenetic alterations in the regulatory processes associated with hematologic malignancies, including the inactivation of tumor suppressor genes and the activation of oncogenes (Cai and Levine, 2019). Acute myeloid leukemia (AML) is the most common type of severe hematological malignant tumor with clonal disorder, which is caused by the oncogenic transformation of myeloid progenitor cells and hematopoietic stem cells (Ferrara and Schiffer, 2013). AML is a severe disease that leads to bone marrow related complications, such as infection, anemia, or bleeding, with a low survival rate and poor prognosis (Cheung et al., 2019).

The E26 transformation-specific (ETS) protein family is the largest family of transcription factors. ERG (ETS-related gene) is a member of the ETS family that regulates endothelial apoptosis and angiogenesis through VE-cadherin (Birdsey et al., 2008). ERG plays a crucial role in regulating the normal function of hematopoietic stem cells and maintaining the number of platelets in peripheral blood in the hematopoietic system. ERG is essential for the self-renewal of hematopoietic stem cells during stress induced hematopoiesis in mice (Ng et al., 2011). Haptad regulated stem cell enhancers enhanced ERG transcription and promoted AML and T-ALL (acute lymphoblastic leukemia) (Thoms et al., 2011; Diffner et al., 2013). ERG promoted expansion and recapitulated molecular signatures of leukemias with high ERG in cord blood progenitors (Tursky et al., 2015). ERG and gene translocation produce fusion gene products that lead to leukemia. ERG has been reported to be a megakaryocytic oncogene, and the fusion oncoprotein FUS-ERG targets key hematopoietic regulators and modulates the ATRA (all-trans retinoic acid) signaling pathway in *t*(16;21) AML (Kong et al., 1997; Salek-Ardakani et al., 2009; Sotoca et al., 2016). There are other types of ERG alterations associated with AML, such as chromosomal translocation *t*(*X*;21)(q25–26; q22), which generates a fusion protein of two ETS family members, ELF4 and ERG (Moore et al., 2006). The high expression of ERG is closely associated with different types of hematological malignancies, affecting the overall and disease-free survival rates of patients (Tsuzuki et al., 2011). ERG is modified by phosphorylation, acetylation and methylation, and the regulation of its epigenetic state might be the strategy to target ERG in AML therapy (Martens, 2011). However, the roles of ERG in the pathogenesis of AML and the associated molecular mechanism remain unclear.

Small ubiquitin-like modifier (SUMO) modification is a post-translational modification (PTM) of proteins in eukaryotes (Agbor et al., 2011). There are three forms of SUMO protein in the human body that can be covalently attached to the lysine residues of target proteins (Varejao et al., 2020). SUMOylation is a dynamic process, and the regulation of the SUMO uncoupling from target proteins by SENP (SUMO/Sentrin-specific protease) is central to the SUMOylation process, which ensures the plasticity of protein-protein interaction networks (Lima and Reverter, 2008). There are six mammalian SENPs that belong to three different subfamilies, and some SENPs can also act as maturation processing enzymes of the SUMO precursors (Kunz et al., 2016). SUMOylation plays an important regulatory role in maintaining normal cell activities. We previously showed that SENP2-null embryos exhibit a cardiac developmental defect and do not survive to birth, as SENP2 regulates the binding of polycomb complex to H3K27me3 (Kang et al., 2010). SENP2 is also an important regulator of myostatin-mediated myogenesis (Qi et al., 2014b) and sudden unexplained death in epilepsy (Qi et al., 2014a; Wu et al., 2016; Chen et al., 2018). SUMOylation abnormalities are closely associated with the occurrence and development of many human diseases, including hematological tumors (Boulanger et al., 2019). SUMOylation participates in the response to chemotherapy in AML, particularly through its ability to regulate gene and protein expression (de The et al., 2017). However, whether the ERG protein is modified by SUMO and the role of ERG SUMOylation in AML remains unknown.

In the present study, we show that ERG promotes the proliferation and inhibits the differentiation of AML cells. ERG was observed to be SUMOylated at multiple sites, while SENP2 specifically deconjugates SUMO proteins from ERG. ERG SUMOylation was observed to inhibit the ubiquitination-mediated degradation of ERG to maintain its stability. In addition, PML was shown to recruit and promote the SUMOylation of ERG, and As_2_O_3_ was demonstrated to inhibit ERG expression in AML cells. The SUMO mutant ERG exhibited a decreased capacity to promote the proliferation and inhibit the differentiation of AML cells. These results provide a good foundation to better understand the role and molecular mechanism of ERG SUMOylation in the development of AML, and provide a new treatment target for clinical treatment of AML.

## Materials and Methods

### Cell Culture and Treatment

HEK293T cells were cultured in DMEM (Gibco) medium, and HL60, K562, THP1, and U937 cells were cultured in RPMI (Gibco) medium containing 10% fetal bovine serum (FBS, Gibco), 100 units/ml penicillin and 100 μg/ml streptomycin and maintained at 37°C in 5% CO_2_ atmosphere. Cells were transiently transfected using Lipofectamine 2000 (Invitrogen), according to the manufacturer’s instructions. For protein degradation assay, transfected cells were treated with 50 μg/mL cycloheximide (CHX, Sigma) or 10 μM MG132 (Sigma) for different periods of time, and the whole cell lysates (WCL) were analyzed by Western blotting. 20 μM of 2-D08 (Sigma) was applied for 24 h to inhibit the SUMOylation pathway in cells. The indicated cells were treated with 0.1 μM As_2_O_3_ for 12 h, and then the WCL were analyzed by Western blotting.

### Plasmid Construction

Eukaryotic expression plasmids RGS-SENP2 wild type, RGS-SENP2 mutant, HA-SUMO1, HA-SUMO2 and HA-Ubiquitin were previously described (Kang et al., 2010; Qi et al., 2014a, b). The Flag-ERG, sh-ERG, and pCDH-ERG plasmids were constructed by standard PCR-based strategies, and SUMO site mutants were generated using the Quick-change site-directed mutagenesis kit (TianGen) with indicated primers (Supplementary Table 1). All cDNAs were completely sequenced (Supplementary Table 2).

### Generation of the Lentiviral System

ETS-related gene stably knockdown or overexpression cell lines were generated using a lentiviral system (System Biosciences). The Virus was generated in HEK293TN cells by transfecting the pGreen-Puro, packaging (psPAX2), and envelope (pMD2) plasmids. Cell culture media was collected 48 h after transfection and immediately transferred to target cells in the presence of polybrene (Sigma). The transduced cells were selected with puromycin for 48 h. Real-time PCR or Immunoblotting (IB) was used to detect the efficacy of knockdown.

### RNA Isolation and Real-Time PCR

For quantitative analysis of gene expression, total RNA was extracted using the RNeasy protocol (Qiagen) from cultured or transfected cells. RNA was treated with 20 U/ml DNase (Promega) for 30 min, and the concentration was determined by measuring absorbance at 260 nm. Equal RNA levels were used to generate complementary DNA using the high-capacity cDNA reverse transcription protocol (Takara). Quantitative real-time PCR was then performed using reaction mixtures of cDNA, indicated primers (Supplementary Table 3), and SYBR Green reagent (Takara) with the ABI StepOne system (PerkinElmer). PCR was done in triplicate, and standard deviations representing experimental errors were calculated. All data were analyzed using ABI PRISM SDS 2.0 software (PerkinElmer). This software, which is coupled to the instrument, allows the determination of the threshold cycle that represents the number of the cycle where the fluorescence intensity is significantly above the background fluorescence intensity. β-Actin was used for normalization and the results were presented as 2^deltaCT (Control-Target) to indicate relative differences in RNA levels.

### Western Blotting and Immunoprecipitation

The protein of transfected cells was extracted and subsequently homogenized on ice in lysis buffer with protease inhibitors (Targetmol). Total protein levels were quantified using the BCA assay (Pierce). Equal protein amounts were separated by electrophoresis and transferred to PVDF membranes. Membranes were blocked, incubated overnight with primary antibodies, washed, incubated with secondary antibody coupled to peroxidase, and detected protein levels with chemiluminescence system (Tanon) after additional washing steps. For co-immunoprecipitation (co-IP) experiments, cell lysates were incubated overnight with antibodies. Antibody-bound protein complexes were captured by the addition of protein A agarose and incubated. Protein A agarose was pelleted by centrifugation, and the IP protein complex was eluted using SDS-PAGE sample buffer and western blotting with antibodies (Supplementary Table 4).

### MTT Detection of Cell Proliferation

Cells were seeded at a density of 3000 cells/well in 96-well plates and incubated in the culture medium. After 24, 48, or 72 h, the total cell number was measured using the 3-(4,5-dimethylthiazol-2-yl)-2,5-diphenyltetrazolium bromide (MTT, Sigma) according to the manufacturer’s protocol. Briefly, 15 μL of 5 mg/mL MTT solution was added to 150 μL culture medium. For the suspended cells, centrifuge and carefully aspirate the culture medium to discard. After 4 h incubation at 37°C, 200 μL dimethyl sulfoxide (DMSO, Sigma) was added to each well. The absorbance was measured using a multimode microplate reader (Thermo) at 490 nm.

### Giemsa Assay

Indicated cells were resuspended in PBS, and drop an appropriate amount onto the front of the slide, and take another slide with a smooth edge as a push slide. Place one end of it in front of the droplet and move it back to touch the droplet to make the droplet even scattered at the contact between the pusher and the slide. Then, at an angle of 30–40°, push it out smoothly to the other end, and place the slide to dry naturally. Cells were fixed in 4% paraformaldehyde (PFA, Sigma) for 5 min and air dry, and stained with Giemsa stain solution (Sigma) for 30 min. The slice was rinsed in PBS and air dry for evaluation by microscopy (Zeiss).

### Immunocytochemistry

Cells transfected with indicated plasmids were grown on cover slips. And 48 h later, cells were washed with PBS, fixed with 4% PFA and permeabilized with 0.5% Triton X-100 in PBS and then fixed with antibodies. Non-specific antibody binding was minimized by treatment with 5% donkey serum in PBS for 30 min at room temperature. Primary antibodies were diluted in 0.1% Triton X-100 in PBS and incubated with the cells for 1 h at 37°C. Cells were washed three times in PBS and then incubated for 1 h at room temperature with secondary antibodies Alexa Fluro 488 or 546 fluorophores (Invitrogen). The cells were then washed three times in PBS and mounted using the anti-fade mounting solution (Invitrogen), and then examined by confocal laser scanning microscopy (Zeiss).

### Flow Cytometry

Cells were isolated and blocked with 1% BSA in PBS for 10 min at room temperature. Fixed and blocked cells were incubated with APC-conjugated anti-CD11b monoclonal antibody (BD PharMingen) for 30 min on ice, and cells were washed three times with 1% BSA in PBS. Staining was analyzed using a FACS Calibur instrument (BD PharMingen).

### Quantification and Statistical Analysis

All data were presented as mean ± S.E.M. or SD at least three separate experiments. Differences between groups were evaluated by the Student’s *t*-test for two-group comparisons, and one-way ANOVA followed by Dunnett’s or Tukey’s test or two-way ANOVA followed by Bonferroni’s test for multiple comparisons among more than two groups. The variance was similar between the groups that were being statistically compared. Statistical significance was defined as *p* < 0.05 (^∗^*p* < 0.05, ^∗∗^*p* < 0.01, and ^∗∗∗^*p* < 0.001).

## Results

### ERG Is Highly Expressed and Closely Correlated With AML

**FIGURE 1:**
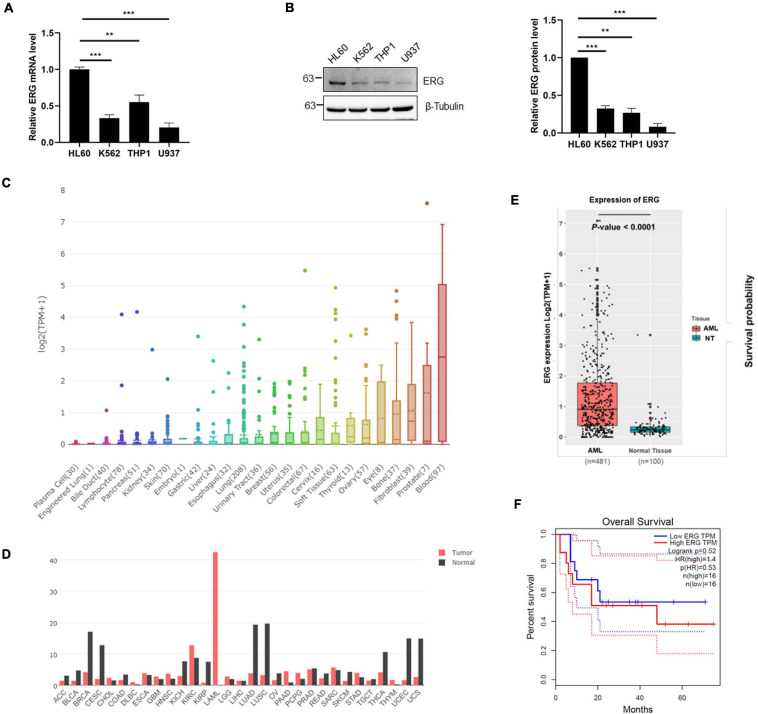
ETS-related gene (ERG) is highly expressed in AML cells and patients. **(A)** Expression levels of ERG transcripts in various myeloid leukemia cells. The expression levels of ERG transcripts in various myeloid cells were measured by real-time PCR and normalized to HL60 cells (*n* = 3 repeats/group, one-way ANOVA, **p < 0.01, ***p < 0.001). **(B)** Expression levels of ERG protein in various myeloid cells. The cell lysates from various myeloid leukemia cells were detected by IB with anti-ERG and anti-β-tubulin antibodies (left). The results of quantitative analysis of Western blot are shown in the right panel (*n* = 3 repeats/group, one-way ANOVA, **p < 0.01, ***p < 0.001). **(C)** The expression levels of ERG transcripts in various tissues in human were analyzed (depmap.org). **(D)** The expression levels of ERG transcripts in various tumor samples were compared to that in paired normal tissues (http://gepia.cancer-pku.cn/). **(E)** RNA-seq data was downloaded from the TCGA leukemia dataset, and the mRNA expression levels of ERG in AML and normal tissues were analyzed by R language. **(F)** ERG overexpression is not associated with prognosis. Patients whose ERG expression levels were one normalized standard deviation above and below the mean level were grouped as high ERG and low ERG, respectively.

To investigate the role of ERG in leukemia, we examined the expression level of ERG in multiple leukemia cell lines, including acute promyelocytic leukemia cell HL60, chronic myelogenous leukemia cell K562, acute monocytic leukemia cell THP1, and histiocytic lymphoma leukemia cell U937. The qPCR results indicated that the ERG transcripts were detectable in these cell lines, with the highest levels observed in HL60 cells (Figure 1A). We next performed immunoblotting (IB) analysis, the results of which showed that these cell lines also showed high ERG protein levels, with the highest levels observed in HL60 cells (Figure 1B), suggesting that ERG is highly expressed in AML cells at the mRNA and protein levels. DepMap database (depmap.org) analysis showed that ERG is expressed in many human organs, with the highest levels observed in the blood (Figure 1C), indicating that ERG plays a crucial role in the circulatory system. We compared the gene expression profiles of ERG in all tumor samples and corresponding normal tissues using the GEPIA database^1^, and found decreased ERG expression in some tumors, with tumor samples of AML patients exhibiting much higher ERG levels than normal tissues (Figure 1D). We further analyzed the RNA-seq data downloaded from the TCGA leukemia dataset, and the results showed that the ERG expression was significantly upregulated in AML patients compared with normal tissues (Figure 1E). The results showed a wide range of expression levels of ERG in AML, which may due to multiple subtypes of AML. To determine the clinical significance of ERG expression, we analyzed the ERG expression levels and overall survival of AML patients using the GEPIA database (quartile cutoff). The results showed that the overall survival had no significant difference between AML patients with high ERG levels and low ERG levels (Figure 1F), which my due to too few numbers of AML patients. Taken together, these results suggested that high ERG levels are closely correlated with AML.

### ERG Promotes the Proliferation and Inhibits the Differentiation of Leukemia Cells

**FIGURE 2:**
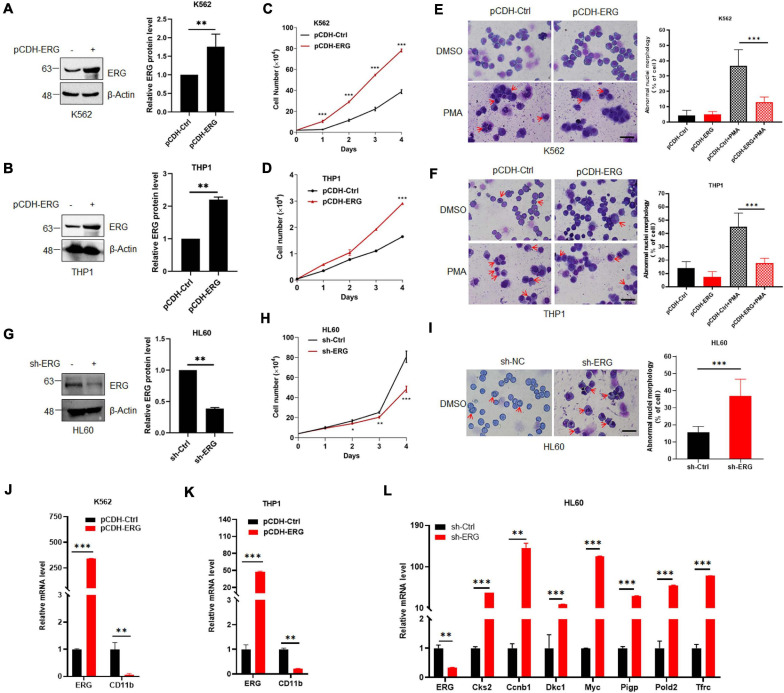
ETS-related gene promotes myeloid leukemia cell proliferation and inhibits cell differentiation. **(A,B)** ERG was stably transfected into K562 **(A)** and THP1 **(B)** cells. pCDH-ERG or control lentivirus was transfected into K562 and THP1 cells, and the cell lysates of stably constructed cells were detected by IB with anti-ERG and anti-β-Actin antibodies (left). The results of quantitative analysis of Western blot are shown in the right panel (*n* = 3 repeats/group, Student’s *t*-test, **p < 0.01). **(C,D)** ERG overexpression promoted the proliferation of K562 **(C)** and THP1 **(D)** cells. Growth curves of ERG overexpression or control cells were constructed from daily quantification of cell numbers (*n* = 3 repeats/group, one-way ANOVA, ***p < 0.001). **(E,F)** ERG overexpression inhibited the PMA induced differentiation of K562 **(E)** and THP1 **(F)** cells. ERG overexpression or control cells were treated with DMSO or PMA, and the cell differentiation was analyzed by Giemsa staining (left). The results of quantitative analysis of differentiated cells are shown in the right panel (*n* = 3 repeats/group, two-way ANOVA, ***p < 0.001). The scale bar is 100 μm. **(G)** ERG was stably knockdown in HL60 cells. sh-ERG or control lentivirus was transfected into HL60 cells, and the cell lysates of stably constructed cells were detected by IB with anti-ERG and anti-(β-Actin antibodies (left). The results of quantitative analysis of Western blot are shown in the right panel (*n* = 3 repeats/group, Student’s *t*-test, **p < 0.01). **(H)** ERG knockdown inhibited the proliferation of HL60 cells. Growth curves of ERG knockdown or control cells were constructed from daily quantification of cell numbers (*n* = 3 repeats/group, one-way ANOVA, *p < 0.05, **p < 0.01, ***p < 0.001). **(I)** ERG knockdown promoted the differentiation of HL60 cells. ERG knockdown or control cells were cultured and the cell differentiation was analyzed by Giemsa staining (left). The results of quantitative analysis of differentiated cells are shown in the right panel (*n* = 3 repeats/group, Student’s *t*-test, ***p < 0.001). The scale bar is 100 μm. **(J,K)** The mRNA level of CD11b was decreased after ERG overexpression in K562 **(J)** and THP1 cells **(K)**. The expression levels of ERG and CD11b transcripts in pCDH-ERG stably transfected cells were measured by real-time PCR and normalized to pCDH-Ctrl cells (*n* = 3 repeats/group, two-way ANOVA, **p < 0.01, ***p < 0.001). **(L)** The mRNA level of ERG target genes was increased after ERG knockdown in HL60 cells. The expression levels of ERG and target genes transcripts in sh-ERG stably transfected HL60 cells were measured by real-time PCR and normalized to sh-Ctrl cells (*n* = 3 repeats/group, two-way ANOVA, **p < 0.01, ***p < 0.001).

As shown in Figure 1B, the ERG protein levels in K562 and THP1 were much less than that in HL60 cells. To elucidate the specific biological functions of ERG in leukemia cells, we stably overexpressed of ERG in K562 and THP1 cells using a lentiviral-based approach (Figures 2A,B). The results showed that ERG overexpression significantly accelerated the proliferation of K562 and THP1 cells (Figures 2C,D). To elucidate the role of ERG in cell differentiation, cells were incubated with DMSO or 3 mM PMA for 24 h, after which Giemsa staining was performed to assess the morphology of the differentiated cells. The staining results showed that PMA induced the differentiation of K562 and THP1 cells, whereas ERG overexpression significantly inhibited cell differentiation (Figures 2E,F). Since the ERG protein levels in HL60 were much higher than that in other cells (Figure 1B), to further elucidate the crucial role of ERG in AML cells, shRNA was used to knockdown ERG expression in HL60 cells (Figure 2G). The results from 4 days of counting cells showed that ERG knockdown significantly inhibited the proliferation of HL60 cells (Figure 2H). The Giemsa staining results showed that ERG knockdown affected the morphology and promoted the differentiation of HL60 cells (Figure 2I). CD11b is the major differentiation marker of AML cells, as expected, the mRNA level of CD11b was dramatically decreased after ERG overexpression in K562 and THP1 cells (Figures 2J,K). It was reported that ERG performed its function through regulation of Myc and its target genes, including Cks2, Ccnb1, Dkc1, Pigp, Pold2, and Tfrc. We furthered detected the mRNA levels of these genes, and the results showed that the expression levels of these genes were significantly increased after ERG knockdown in HL60 cells (Figure 2L). Taken together, these results show that ERG promotes the proliferation and inhibits the differentiation of leukemia cells, indicating that ERG plays an important role in regulating the proliferation and differentiation of leukemia cells.

### ERG Is SUMOylated and Mainly Modified by SUMO2

**FIGURE 3:**
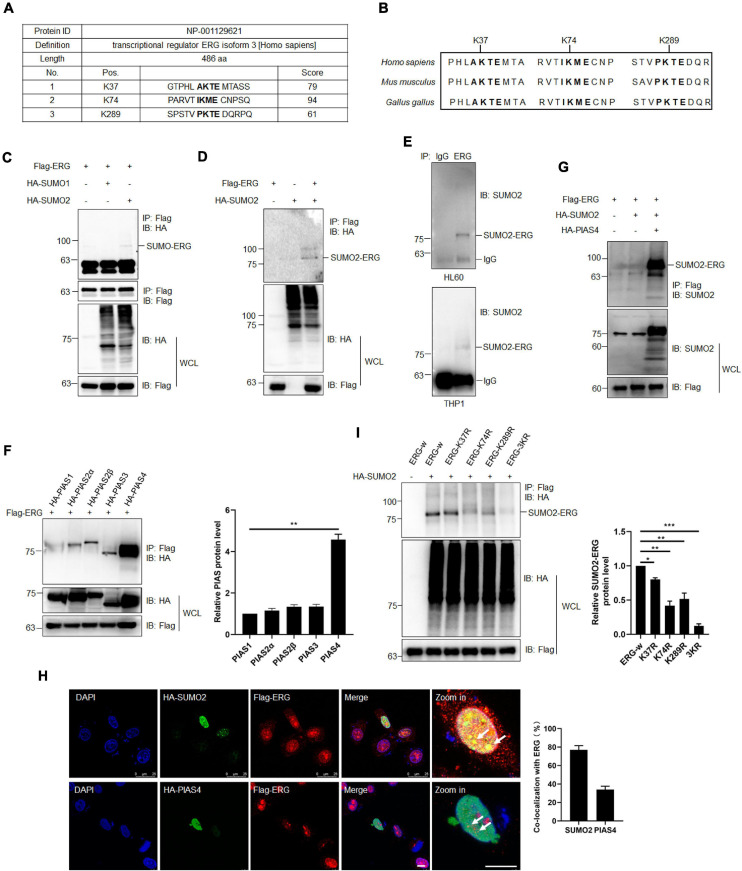
ETS-related gene SUMOylation is dynamically regulated by SUMO2 and PIAS4. **(A)** The predicted SUMOylation sites of ERG by online software (www.abcepta.com/sumoplot). **(B)** The potential SUMOylation sites were highly conserved in various species. **(C)** ERG is mainly SUMOylated by SUMO2 in HEK293T cells. The indicated plasmids were transfected into HEK293T cells, and the cell lysates were detected by IP with anti-Flag antibody, followed by IB with anti-HA antibody. The whole cell lysates (WCL) were detected by IB with anti-Flag and anti-HA antibodies. **(D)** SUMO2 promoted the SUMOylation of ERG. The indicated plasmids were transfected into HEK293T cells, and the cell lysates were detected by IP with anti-Flag antibody, followed by IB with anti-HA antibody. The WCL were detected by IB with anti-Flag and anti-HA antibodies. **(E)** ERG was SUMOylated by endogenous SUMO2. The IP with IgG or ERG from HL60 (upper) and THP1 (bottom) cell lysates were detected by IB with anti-SUMO2 antibody. **(F)** PIAS4 was the major E3 ligase of ERG. The indicated plasmids were transfected into HEK293T cells, and the IP with Flag from cell lysates were detected by IB with anti-HA antibody. The WCL were detected by IB with anti-HA or anti-Flag antibodies (left). The results of quantitative analysis of IP-western are shown in the right panel (*n* = 3 repeats/group, one-way ANOVA, **p < 0.01). **(G)** PIAS4 enhanced the SUMOylation of ERG. The indicated plasmids were transfected into HEK293T cells, and the IP with Flag from cell lysates were detected by IB with anti-SUMO2 antibody. The WCL were detected by IB with anti-SUMO2 or anti-Flag antibodies. **(H)** ERG colocalized with SUMO2 and PIAS4 in the nucleus. The indicated plasmids were transfected into HEK293T cells, and cells were harvested for immunofluorescence staining with indicated antibodies (left). The results of quantitative analysis of co-localization are shown in the right panel (*n* = 3 repeats/group, Student’s *t*-test). DAPI (blue) was used to show nuclei. The scale bar is 10 μm. **(I)** K37, K74, and K289 were the major SUMOylation sites of ERG. Wild-type or mutant Flag-ERG and HA-SUMO2 were transfected into HEK293T cells, and the IP with Flag from cell lysates were detected by IB with anti-HA antibody. The WCL were detected by IB with anti-HA or anti-Flag antibodies (left). The results of quantitative analysis of IP-western are shown in the right panel (*n* = 3 repeats/group, one-way ANOVA, *p < 0.05, **p < 0.01, ***p < 0.001).

As increasing evidence has shown that the SUMOylation of target proteins plays a crucial role in leukemia (Boulanger et al., 2019), we assessed whether ERG is modified by SUMO. Using^2^ SUMOplot^TM^, ERG was highly likely to be SUMOylated (Figure 3A), including K37, K74, and K289, with these three sites being evolutionarily conserved in different species (Figure 3B). To determine whether ERG is SUMOylated in human cells, we transfected plasmids encoding ERG, SUMO1, or SUMO2 into HEK293T cells, and the results showed that ERG was primarily conjugated and modified by exogenous SUMO2 (Figures 3C,D). We further identified the SUMOylation status of ERG in AML cells, and the results showed that ERG was modified by endogenous SUMO2 in both HL60 and THP1 cells (Figure 3E). Taken together, these results indicated that ERG was SUMOylated by SUMO2 in both exogenous and endogenous conditions.

To further study the SUMO-mediated regulation of ERG, we subsequently attempted to identify the SUMO E3 ligase that catalyzes ERG SUMOylation. Since PIAS family members are major E3 ligases, we transfected different PIAS plasmids with ERG into HEK293T cells, and the co-IP assay results revealed that PIAS4 was the primary SUMO E3 ligase to interact with ERG (Figure 3F). Consistent with the binding data, PIAS4 also dramatically enhanced the SUMOylation of ERG (Figure 3G). These results indicate that PIAS4 is a specific E3 ligase catalyzing ERG SUMOylation.

To determine the subcellular localization of ERG, specific plasmids were transfected into HEK293T cells, and immunofluorescence (IF) staining results showed that ERG colocalized with PIAS4 and SUMO2 in the nucleus of HEK293T cells (Figure 3H). Bioinformatics analysis of ERG led to the identification of three potential consensus motifs for SUMO conjugation, and they include K37, K74, and K289 (Figure 3A). Subsequently, we generated ERG plasmid constructs with single or triple SUMOylation site mutations to identify ERG SUMOylation sites. The mapping results revealed that the K37, K74, and K289 residues were major SUMOylation sites of ERG (Figure 3I). Taken together, these results indicate that ERG is SUMOylated *in vitro* and *in vivo* at three major SUMOylation sites.

### SENP2 Specifically Deconjugates SUMO2 From ERG

**FIGURE 4:**
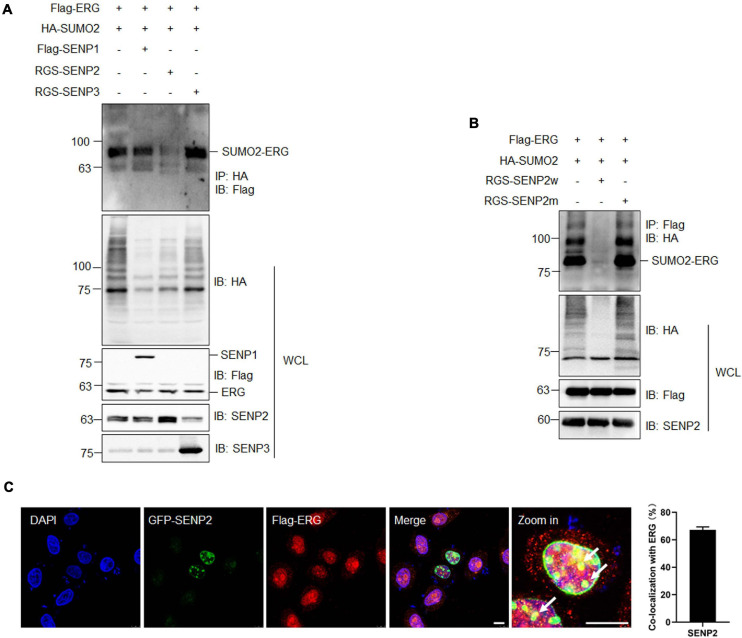
SENP2 deconjugates SUMO2 from SUMOylated ERG. **(A)** SENP2 specifically deconjugated the SUMOylation of ERG. The indicated plasmids were transfected into HEK293T cells, and the IP with HA from cell lysates were detected by IB with anti-Flag antibody. The WCL were detected by IB with anti-HA, anti-Flag, anti-SENP2, or anti-SENP3 antibodies. **(B)** SENP2 wild type, but not catalytic mutant, specifically deconjugated the SUMOylation of ERG. The indicated plasmids were transfected into HEK293T cells, and the IP with Flag from cell lysates were detected by IB with anti-HA antibody. The WCL were detected by IB with anti-HA, anti-Flag, or anti-SENP2 antibodies. **(C)** ERG colocalized with SENP2 in the nucleus. The indicated plasmids were transfected into HEK293T cells, and cells were harvested for immunofluorescence staining with indicated antibodies (left). The results of quantitative analysis of co-localization are shown in the right panel (*n* = 3 repeats/group, Student’s *t*-test). DAPI (blue) was used to show nuclei. The scale bar is 10 μm.

SUMOylation is catalyzed by several enzymes and can be reversed by a family of SENPs. To identify the specific SENP associated with the deconjugation of ERG SUMOylation, three SENP members were cotransfected with ERG and SUMO2, and the co-IP results showed that SENP2 specifically reversed ERG SUMOylation (Figure 4A). Furthermore, wild-type SENP2, but not catalytic mutant SENP2, deconjugated SUMO-modified ERG (Figure 4B). To determine the subcellular localization of ERG, specific plasmids were transfected into HEK293T cells, and immunofluorescence (IF) staining results showed that ERG colocalized with SENP2 in the nucleus of HEK293T cells (Figure 4C). Taken together, these results indicate that SENP2 interacts with ERG to deconjugate SUMO modifications, and SUMO2 and SENP2 together dynamically regulate the SUMOylation status of ERG.

### PML Promotes ERG SUMOylation, Inhibiting the Ubiquitination and Enhancing ERG Stability

**FIGURE 5:**
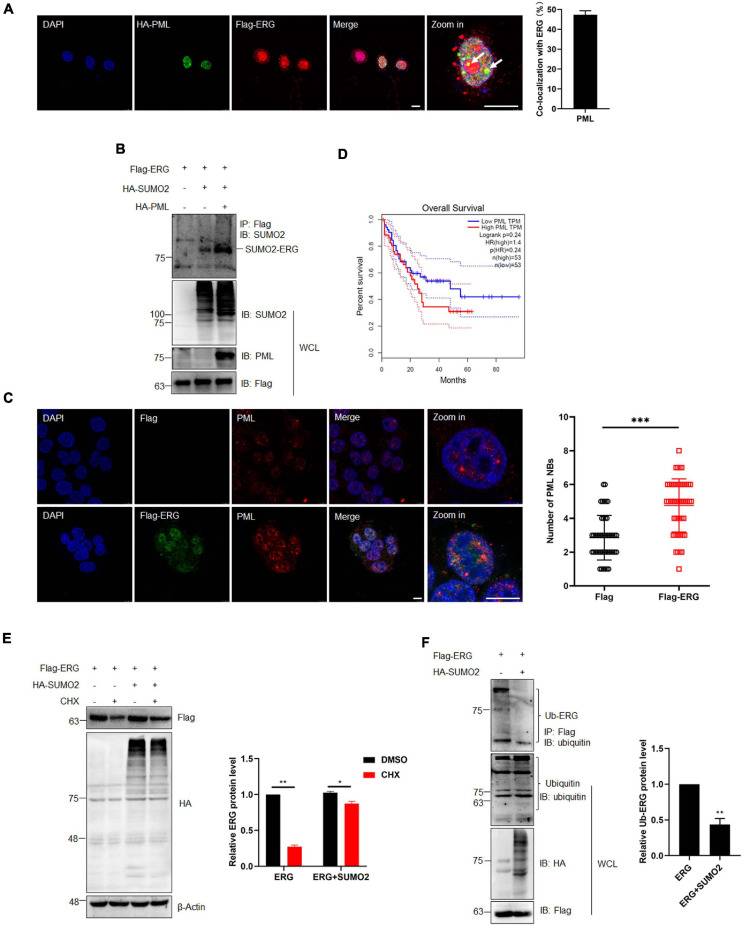
PML recruited and enhanced the SUMOylation of ERG, and SUMOylation promotes the stability of ERG. **(A)** The localization of endogenous ERG and PML in the nucleus. HEK293T cell were cultured for immunocytochemistry with ERG (red) and PML (green) specific antibodies (left). The results of quantitative analysis of co-localization are shown in the right panel (*n* = 3 repeats/group, Student’s *t*-test). DAPI (blue) was used to show nuclei. The scale bar is 10 μm. **(B)** PML enhances the SUMOylation of ERG. The indicated plasmids were transfected into HEK293T cells, and the IP with FLAG from cell lysates were detected by IB with anti-SUMO2 antibody, and the WCL were detected by IB with anti-SUMO2, anti-PML, or anti-FLAG antibodies. **(C)** ERG promotes the formation of PML NBs. Flag-ERG and HA-PML plasmids were transfected into HEK293T cells, and harvested for immunocytochemistry staining with Flag (red) and PML (green)-specific antibodies (left). The results of quantitative analysis of co-localization are shown in the right panel (*n* = 3 repeats/group, Student’s *t*-test, ***p < 0.001). DAPI (blue) was used to show nuclei. The scale bar is 10 μm. **(D)** PML overexpression is not associated with prognosis. Patients whose ERG expression levels were one normalized standard deviation above and below the mean level were grouped as high ERG and low ERG, respectively. **(E)** SUMOylation promotes the stability of ERG. The indicated plasmids were transfected into HEK293T cells, and cells were treated with CHX for 6 h. The transfected cells were harvested and the cell lysates were detected by IB with anti-HA, anti-Flag, or anti-β-actin antibodies (left). The results of quantitative analysis of Western blot are shown in the right panel (*n* = 3 repeats/group, two-way ANOVA, *p (<0.05, ***p (<0.001). **(F)** SUMOylation inhibited the ubiquitination of ERG. FLAG-ERG and HA-SUMO2 plasmids were transfected into HEK293T cells, and the IP with Flag from cell lysates were detected by IB with anti-ubiquitin antibody. The WCL were detected by IB with anti-ubiquitin, anti-HA, or anti-Flag antibodies (left). The results of quantitative analysis of Western blot are shown in the right panel (*n* = 3 repeats/group).

PML acts as the master organizer and scaffold of PML NBs and recruits proteins for SUMO modification, with the PML-RARα fusion protein formed by PML being an important pathogenic factor of APL (Singh et al., 2017; Voisset et al., 2018). Therefore, we transfected ERG and PML into HEK293T cells and assessed their subcellular localization. The IF staining results showed that ERG localized to the nuclear rim, while PML localized to the nucleus, with some ERG and PML localizing (Figure 5A). To assess the role of PML in the SUMOylation of ERG, HEK293T cells were transfected with ERG, SUMO2, and PML plasmids, and the co-IP results showed that PML significantly promoted ERG SUMOylation (Figure 5B). To elucidate whether ERG affects the formation of PML NBs, we transfected control or ERG plasmids into HEK293T cells and assessed the levels of endogenous PML NBs. The IF staining results showed that exogeneous ERG dramatically increased the number of PML NBs (Figure 5C). To determine the clinical significance of ERG induced PML expression, we analyzed the PML expression levels and overall survival of AML patients using the GEPIA database (quartile cutoff). The results showed that the overall survival had no significant difference between AML patients with high PML levels and low PML levels (Figure 5D), suggesting that PML level *per se* is not associated with the prognosis of AML patients. These results showed that PML promotes ERG SUMOylation and that ERG promotes the formation of PML NBs, indicating that a feedback regulatory loop is established between ERG and PML, which may be participated in the pathogenesis of AML.

SUMOylation regulates multiple cellular processes, including protein stability (Yeh, 2009). To determine whether SUMOylation has any effect on the stability of ERG, we transfected HEK293T cells with ERG and SUMO2 plasmids and harvested the cellular proteins for assessment by western blotting after treatment with 10 μM CHX for 6 h. The results showed that CHX treatment promoted ERG protein degradation, whereas SUMO2 significantly postponed ERG protein degradation (Figure 5E), indicating that SUMOylation enhances ERG protein stability. Protein degradation is primarily mediated by the ubiquitin-proteasome pathway. To determine whether the SUMOylation of ERG affects its ubiquitination, HEK293T cells were transfected with ERG and SUMO2 plasmids for co-IP experiments. The results showed that SUMO2 dramatically reduced the ubiquitination of ERG (Figure 5F), indicating that ERG SUMOylation inhibits the ubiquitin-proteasome mediated degradation of ERG. Taken together, these results show that PML promotes ERG SUMOylation, inhibiting the ubiquitination-mediated degradation.

### Arsenic Trioxide and the Inhibition of SUMOylation Promotes AML Cell Differentiation

**FIGURE 6:**
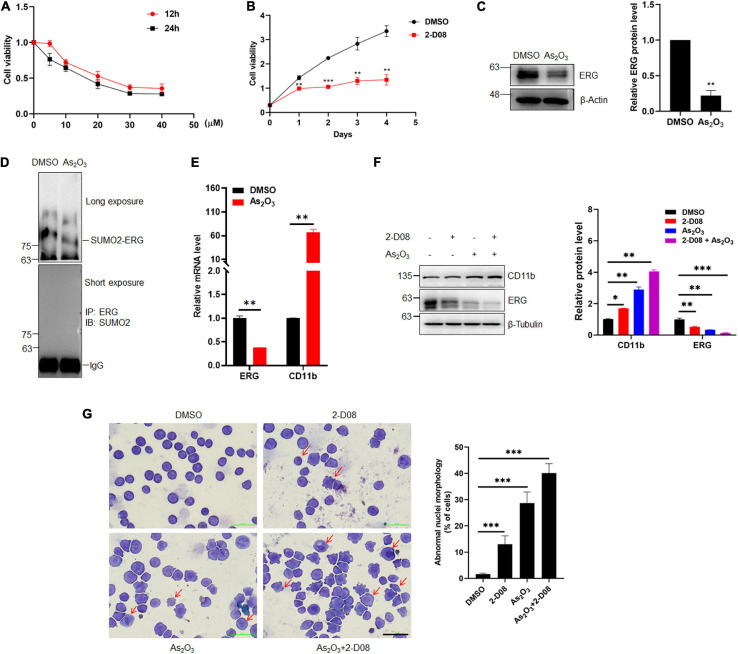
Arsenic trioxide and SUMO inhibitor inhibited the expression of ERG and promoted cell differentiation of HL60 cells. **(A)** 2-D08 suppressed the viability of HL60 cells in a concentration-dependent manner. HL60 cells were incubated with the indicated concentrations of 2-D08 or DMSO for 12 or 24 h and cell viability was measured using MTT assay (*n* = 3 repeats/group, one-way ANOVA). **(B)** 2-D08 suppressed the viability of HL60 cells in a time-dependent manner. HL60 cells were incubated with 20 μM 2-D08 or DMSO for different periods of time and cell viability was measured using MTT assay (*n* = 3 repeats/group, one-way ANOVA, **p < 0.01, ***p < 0.001). **(C)** Arsenic trioxide decreased the expression level of ERG in HL60 cells. HL60 cells were treated with As_2_O_3_ for 24 h, and the cell lysates were detected by IB with anti-ERG or anti-β-actin antibodies (left). The results of quantitative analysis of Western blot are shown in the right panel (*n* = 3 repeats/group, Student’s *t*-test, **p < 0.01). **(D)** Arsenic trioxide decreased the SUMOylation level of ERG in HL60 cells. HL60 cells were treated with As_2_O_3_ for 24 h, and the IP with ERG from cell lysates were detected by IB with anti-SUMO2 antibody. **(E)** The mRNA level of CD11b was decreased after As_2_O_3_ treatment in HL60 cells. The expression levels of ERG and CD11b transcripts after As_2_O_3_ treatment in HL60 cells were measured by real-time PCR and normalized to DMSO control cells (*n* = 3 repeats/group, one-way ANOVA, **p < 0.01). **(F)** Arsenic trioxide and 2-D08 enhanced CD11b expression and inhibited ERG expression in HL60 cells. HL60 cells were treated with As_2_O_3_ and 2-D08 for 24 h, and the cell lysates were detected by IB with CD11b, anti-ERG, or anti-β-actin antibodies (left). The results of quantitative analysis of Western blot are shown in the right panel (*n* = 3 repeats/group, two-way ANOVA, *p < 0.05, **p < 0.01, ***p < 0.001). **(G)** Arsenic trioxide and 2-D08 promoted cell differentiation of HL60 cells. HL60 cells were treated with As_2_O_3_ and 2-D08 for 24 h, and the cell morphology was indicated by Giemsa staining (left). The results of quantitative analysis of differentiated cells are shown in the right panel (*n* = 3 repeats/group, one-way ANOVA, ^∗∗∗^*p* < 0.001). The scale bar is 100 μm.

2-D08 is a SUMOylation inhibitor that specifically inhibits the E2 binding enzyme UBC9. To determine whether the inhibition of SUMOylation exhibit anti-cancer effects against human AML cells, an MTT assay was performed to evaluate the HL60 cells treated with 2-D08. The MTT assay results showed that HL60 cell proliferation was significantly suppressed by 2-D08, and this inhibition was enhanced as the 2-D08 concentration and time were increased (Figures 6A,B). As_2_O_3_ is used to degrades mutated NPM1, which eventually leads to AML cell apoptosis, thereby treating myeloid leukemia patients (El Hajj et al., 2015; Martelli et al., 2015). As_2_O_3_ and ATRA act as two clinically effective therapeutic agents for APL through degradation of PML-RARA fused oncoprotein (de The et al., 2012). Therefore, we assessed whether As_2_O_3_ has any effect on ERG protein expression. To this end, HL60 cells were treated with As_2_O_3_ for 24 h, after which cellular proteins were extracted for western blotting analysis. The results showed that ERG protein expression was significantly decreased in HL60 cells after treatment with As_2_O_3_ (Figure 6C). Since ERG was SUMOylated in AML cells, we asked whether As_2_O_3_ has any effect on ERG SUMOylation, and the results showed that As_2_O_3_ decreased the SUMOylation level of ERG (Figure 6D), with increased mRNA level of CD11b (Figure 6E). We further assessed the synergistic effect of As_2_O_3_ and 2-D08 on ERG levels, with the results showing that combined treatment with As_2_O_3_ and 2-D08 decreased ERG protein levels (Figure 6F). The levels of the cell differentiation marker CD11b were also detected, with the results showing that both As_2_O_3_ and 2-D08 significantly increased CD11b levels (Figure 6F). In addition, HL60 cells were treated with arsenic trioxide and 2-D08 for 24 h, after which Giemsa staining was performed to confirm their effects on cell differentiation. The staining results showed no significant difference in the differentiation of HL60 cells after the 2-D08 treatment, whereas their differentiation of HL60 cells was dramatically promoted after the arsenic trioxide treatment (Figure 6G). Taken together, these results show that SUMOylation inhibition decreased the viability of AML cells, and As_2_O_3_ promoted cell differentiation by inhibiting the expression of ERG in AML cells.

### SUMOylation Contributes to the ERG-Mediated Promotion of Cell Proliferation and Inhibition of Cell Differentiation

**FIGURE 7:**
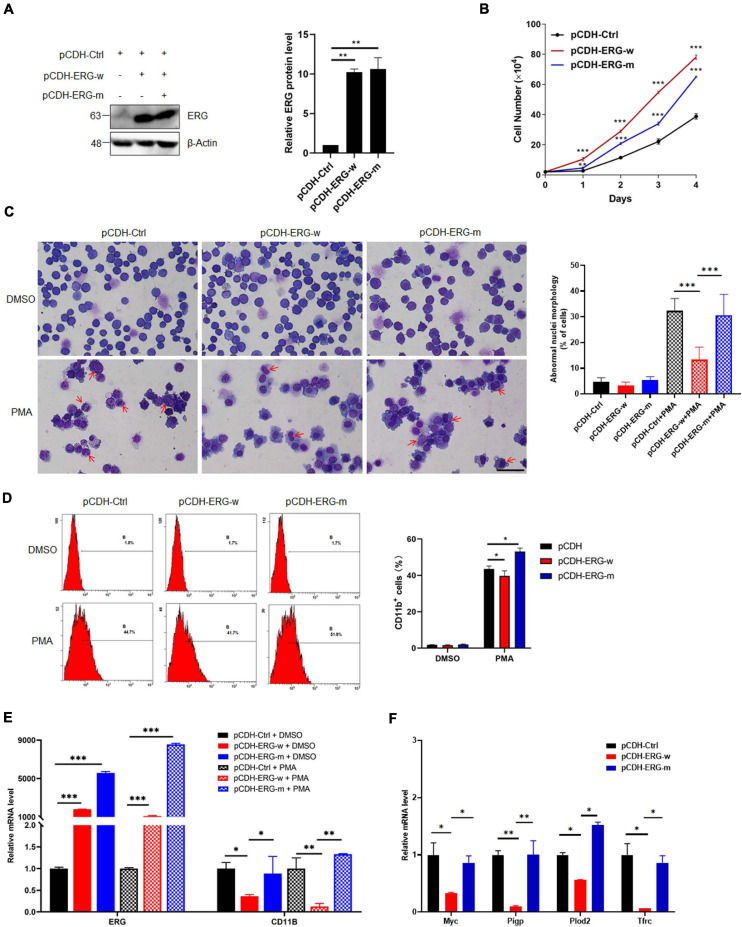
SUMO mutant ERG reversed the increased cell proliferation and decreased cell differentiation by wild-type ERG. **(A)** Wild-type or SUMO mutant ERG was stably transfected into THP1 cells. The indicated lentivirus was transfected into THP1 cells, and the cell lysates of stably constructed cells were detected by IB with anti-ERG and anti-β-Actin antibodies (left). The results of quantitative analysis of Western blot are shown in the right panel (*n* = 3 repeats/group, one-way ANOVA, **p < 0.01). **(B)** Mutant ERG reversed the increased cell proliferation induced by wild-type ERG. Growth curves of wild-type or mutant ERG overexpression or control cells were constructed from daily quantification of cell numbers (*n* = 3 repeats/group, one-way ANOVA, **p < 0.01, ***p < 0.001). **(C)** Mutant ERG promoted the PMA induced morphology change of THP1 cells. Logarithmically growing THP1 cells stably transfected with wild-type or mutant ERG were exposed to DMSO or PMA for 24 h, and the cell morphology was indicated by Giemsa staining (left). The results of quantitative analysis of differentiated cells are shown in the right panel (*n* = 3 repeats/group, two-way ANOVA, ***p < 0.001). The scale bar is 100 μm. **(D)** Mutant ERG reversed the inhibited cell differentiation induced by wild-type ERG. Logarithmically growing THP1 cells stably transfected with wild-type or mutant ERG were exposed to PMA for 24 h. The percentage of cells expressing the monocytic maturation marker CD11b was determined by flow cytometry (Left). The percentage of cells expressing the monocytic maturation marker CD11b was quantified (right, *n* = 3 repeats/group, two-way ANOVA, *p < 0.05). **(E)** Mutant ERG rescued the mRNA level of CD11b that was decreased after wild-type ERG in THP1 cells. The expression levels of ERG and CD11b transcripts in wild-type and mutant pCDH-ERG stably transfected cells were measured by real-time PCR and normalized to pCDH-Ctrl cells (*n* = 3 repeats/group, two-way ANOVA, **p < 0.01, ***p < 0.001). **(F)** Mutant ERG rescued the mRNA level of ERG target genes that were decreased after wild-type ERG in THP1 cells. The expression levels of ERG and target genes transcripts in wild-type and mutant pCDH-ERG stably transfected THP1 cells were measured by real-time PCR and normalized to sh-Ctrl cells (*n* = 3 repeats/group, two-way ANOVA, *p < 0.05, **p < 0.01).

To elucidate the role of ERG SUMOylation in leukemia cells, we generated stable THP1 cells by lentiviral infection with wild-type (ERG-w) or three SUMOylation sites mutant (ERG-m) ERG plasmids. The exogeneous protein expression levels of the wild-type and mutant ERG were detected in THP1 cells by western blotting, and the results showed that both the wild-type and mutant ERG were stably expressed in THP1 cells (Figure 7A). We first assessed the effect of wild-type or mutant ERG on cell proliferation, with the results showing that both wild-type and mutant ERG significantly promoted cell proliferation compared to the control cells. However, cells transfected with the mutant ERG construct exhibited a slower proliferation rate than those transfected with wild-type ERG construct, suggesting that mutant ERG inhibited wild-type ERG induced the proliferation of THP1 cells (Figure 7B).

To determine whether ERG SUMOylation is involved in the differentiation of AML cells, THP1 cells were stably transfected with wild-type or mutant ERG constructs with PMA for 24 h and then analyzed them by Giemsa staining. The results showed that the nucleocytoplasmic ratio of THP1 cells overexpressing mutant ERG was decreased compared to the wild-type ERG group upon PMA treatment, indicating that THP1 cell differentiation was significantly enhanced (Figure 7C). Furthermore, we measured the cell surface expression of the monocytic maturation marker CD11b in these cells by flow cytometry. The results showed that PMA incubation significantly increased CD11b expression in the wild-type ERG, mutant ERG, and control cells (Figure 7D). However, PMA stimulated CD11b expression less potently in the wild-type ERG cells than in the mutant cells (Figure 7D). As expected, the mRNA level of CD11b and ERG target genes were dramatically decreased after wild-type ERG overexpression in THP1 cells (Figures 7E,F). However, the expression levels of these genes were significantly increased after mutant ERG overexpression, comparing to wild-type ERG in HL60 cells (Figures 7E,F). Taken together, these results suggest that mutation of ERG SUMOylation sites reduced the capacity of ERG to induce the proliferation and inhibit the differentiation of AML cells, indicating that the SUMOylation of ERG plays a crucial role in the pathogenesis of AML.

## Discussion

**FIGURE 8:**
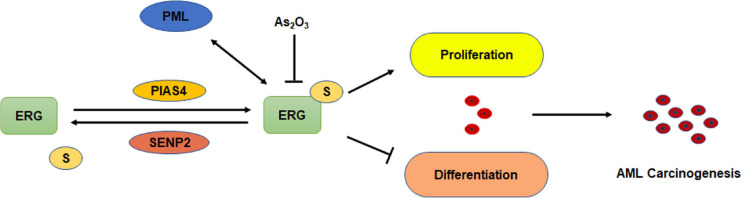
Schematic model depicting the role of SUMOylation of ERG in the regulation of cell proliferation and differentiation of AML cells.

Although ERG plays an important role in the pathogenesis of AML, whether ERG is SUMOylated and the specific regulatory mechanism associated with this activity in the pathogenesis of AML remains unclear. In the present study, we demonstrated that ERG promotes the proliferation and inhibits the differentiation of AML cells. ERG was shown to be SUMOylated at multiple sites, and SENP2 was observed to deconjugate SUMO groups from ERG. SUMOylation inhibited the degradation of ERG via the ubiquitin-proteasome pathway and enhanced ERG stability. Mutation of the SUMOylation sites in ERG partially abolished the influence of ERG on the proliferation and differentiation of AML cells (Figure 8). These findings indicate that the SUMOylation of ERG plays an important role in the pathogenesis of AML.

ERG has previously been reported to be involved in myeloid and lymphoid malignancies and plays a crucial role in the progression of leukemia. A previous study showed that ERG is capable of promoting the development of leukemia and is crucial for its maintenance (Tsuzuki et al., 2011). Furthermore, ERG overexpression drives aberrant megakaryopoiesis, and AKT functions together with ERG to dysregulate megakaryopoiesis and promote AMKL (Stankiewicz and Crispino, 2013). Consistent with these findings, we showed that ERG promotes the proliferation and inhibits the differentiation of AML cells (Figure 2). ERG knockdown attenuated growth in both Down syndrome and non-Down syndrome AMKL (acute megakaryoblastic leukemia) cell lines (Salek-Ardakani et al., 2009), and human leukemia cell lines of various lineage (Tsuzuki et al., 2011). On the other hand, ERG functions as both a transcriptional activator and a repressor in HSCs (hematopoietic stem cells) and coordinately regulates the expression of genes associated with self-renewal and differentiation, indicating a unique role for ERG in normal stem cell biology (Knudsen et al., 2015).

Increasing evidence suggests that SUMOylation may play a major role in the development of the hematopoietic system and AML pathogenesis. In multiple myeloma, many enzymes of the SUMO pathway are overexpressed, and their expression correlates with a lower response to chemotherapies (Driscoll et al., 2010). B-cell lymphomas overexpressing the c-Myc oncogene also overexpress most components of the SUMO pathway and are highly sensitive to SUMOylation inhibition (Boulanger et al., 2019). ERG was reported to be phosphorylated by MAPK/ERK2, and ERG phosphorylation promoted stem cell signatures and cell proliferation (Huang et al., 2016). We observed that ERG was modified by SUMO2, another type of post-translational modification (Figure 3). We demonstrated that ERG SUMOylation contributes to the progression of AML, whereas the SUMO mutant ERG exhibited reduced ability to facilitate the proliferation and differentiation of AML cells (Figure 6). These results indicate that ERG SUMOylation may be a risk factor for leukemia pathogenesis, and SUMO conjugation biomarkers can be used to predict the response of AML patients to chemotherapies (Gatel et al., 2020).

Arsenic trioxide degrades the PML-RARα fusion protein and has been successfully used in the clinic for the treatment of APL (de The et al., 2012). Arsenic promotes remission in APL patients by acting upon several pathways, including the stimulation of differentiation, the induction of apoptosis, and the inhibition of angiogenesis (Tomita et al., 2013). PML plays a key role in the development of leukemia, and recruits proteins for SUMO modification (de The et al., 2017), and we observed that PML promotes the SUMOylation of ERG (Figure 4). Arsenic trioxide binds to PML or PML/RARα and induces SUMO conjugation, and arsenic specifically induces the SUMO-dependent recruitment of ubiquitin E3 RNF4 to PML NBs. Under the action of SUMO-targeted ubiquitin ligase E3, PML is degraded through the proteasome pathway, leading to reduced levels of PML-RARα protein, which is followed by subsequent APL cell apoptosis (Geoffroy et al., 2010). We treated HL60 cells with SUMO inhibitor and As_2_O_3_, and found that they both inhibited ERG expression and promoted cell differentiation (Figure 5). These results indicate that PML enhances the SUMOylation of ERG to maintain its stability, whereas the SUMOylation inhibitor or As_2_O_3_ decreased its stability. The regulation of ERG stability by SUMOylation may be a novel strategy for the treatment of AML.

In summary, in the present study, we have provided evidence that ERG SUMOylation plays a crucial role during AML progression by promoting the growth and inhibiting the differentiation of AML cells. Mutation of the SUMOylation sites in ERG inhibits the proliferation and promotes the differentiation of AML cells. Since ERG is frequently overexpressed in a variety of leukemias, ERG SUMOylation may be an important regulator in the occurrence and development of this disease. The results of the present study provide evidence for further investigation of the molecular mechanism of ERG SUMOylation in the progression of AML and identified a potential target for the clinical treatment of AML.

## Data Availability Statement

The original contributions generated for this study are included in the article/Supplementary Material, further inquiries can be directed to the corresponding authors.

## Ethics Statement

The animal study was reviewed and approved by Institutional Animal Care and Use Committee of Shaanxi Normal University.

## Author Contributions

XC and ZZ conducted RNA isolation and real-time PCR assay. XC and BZ performed Western blotting and immunoprecipitation analysis. XC, YuQ, and HY manipulated the flow cytometry assay. XC and ZZ conducted the immunocytochemistry assay. ZZ and XY preformed MTT assay. XC and ZX produced Giemsa staining. YuQ and YS produced the plasmid construction. WL and CD analyzed the publicly available data sets. XC, ZZ, and QW preformed the quantification and statistical analysis with oversaw of HW and YiQ. HW and YiQ supervised and conceived the project. HS and XW made some suggestions. XC, HW, and YiQ wrote the manuscript with editorial input from ZZ. All authors contributed to the article and approved the submitted version.

## Conflict of Interest

The authors declare that the research was conducted in the absence of any commercial or financial relationships that could be construed as a potential conflict of interest.

## Footnotes

1 http://gepia.cancer-pku.cn/

2 www.abcepta.com/sumoplot

## References

[B1] Agbor T. A. Cheong A. Comerford K. M. Scholz C. C. Bruning U. Clarke A. (2011). Small ubiquitin-related modifier (SUMO)-1 promotes glycolysis in hypoxia. *J. Biol. Chem.* 286 4718–4726. 10.1074/jbc.m110.115931 21123177 PMC3039330

[B2] Birdsey G. M. Dryden N. H. Amsellem V. Gebhardt F. Sahnan K. Haskard D. O. (2008). Transcription factor Erg regulates angiogenesis and endothelial apoptosis through VE-cadherin. *Blood* 111 3498–3506. 10.1182/blood-2007-08-105346 18195090 PMC2275018

[B3] Boulanger M. Paolillo R. Piechaczyk M. Bossis G. (2019). The SUMO pathway in hematomalignancies and their response to therapies. *Int. J. Mol. Sci.* 20:3895. 10.3390/ijms20163895 31405039 PMC6721055

[B4] Cai S. F. Levine R. L. (2019). Genetic and epigenetic determinants of AML pathogenesis. *Semin. Hematol.* 56 84–89. 10.1053/j.seminhematol.2018.08.001 30926095 PMC8961685

[B5] Chen X. Zhang S. Huang J. Dong W. Xiao H. Shao H. (2018). Hyper-SUMOylation of K(+) channels in sudden unexplained death in epilepsy: isolation and primary culture of dissociated hippocampal neurons from newborn mice for subcellular localization. *Methods Mol. Biol.* 1684 63–71. 10.1007/978-1-4939-7362-0_6 29058184

[B6] Cheung E. Perissinotti A. J. Bixby D. L. Burke P. W. Pettit K. M. Benitez L. L. (2019). The leukemia strikes back: a review of pathogenesis and treatment of secondary AML. *Ann. Hematol.* 98 541–559. 10.1007/s00277-019-03606-0 30666431

[B7] de The H. Le Bras M. Lallemand-Breitenbach V. (2012). The cell biology of disease: acute promyelocytic leukemia, arsenic, and PML bodies. *J. Cell Biol.* 198 11–21. 10.1083/jcb.201112044 22778276 PMC3392943

[B8] de The H. Pandolfi P. P. Chen Z. (2017). Acute promyelocytic leukemia: a paradigm for oncoprotein-targeted cure. *Cancer Cell* 32 552–560. 10.1016/j.ccell.2017.10.002 29136503

[B9] Diffner E. Beck D. Gudgin E. Thoms J. A. Knezevic K. Pridans C. (2013). Activity of a heptad of transcription factors is associated with stem cell programs and clinical outcome in acute myeloid leukemia. *Blood* 121 2289–2300. 10.1182/blood-2012-07-446120 23327922

[B10] Driscoll J. J. Pelluru D. Lefkimmiatis K. Fulciniti M. Prabhala R. H. Greipp P. R. (2010). The sumoylation pathway is dysregulated in multiple myeloma and is associated with adverse patient outcome. *Blood* 115 2827–2834. 10.1182/blood-2009-03-211045 19965618 PMC2854429

[B11] El Hajj H. Dassouki Z. Berthier C. Raffoux E. Ades L. Legrand O. (2015). Retinoic acid and arsenic trioxide trigger degradation of mutated NPM1, resulting in apoptosis of AML cells. *Blood* 125 3447–3454. 10.1182/blood-2014-11-612416 25800051

[B12] Ferrara F. Schiffer C. A. (2013). Acute myeloid leukaemia in adults. *Lancet* 381 484–495. 23399072 10.1016/S0140-6736(12)61727-9

[B13] Gatel P. Brockly F. Reynes C. Pastore M. Hicheri Y. Cartron G. (2020). Ubiquitin and SUMO conjugation as biomarkers of acute myeloid leukemias response to chemotherapies. *Life Sci. Alliance* 3:e201900577. 10.26508/lsa.201900577 32303586 PMC7167290

[B14] Geoffroy M. C. Jaffray E. G. Walker K. J. Hay R. T. (2010). Arsenic-induced SUMO-dependent recruitment of RNF4 into PML nuclear bodies. *Mol. Biol. Cell* 21 4227–4239. 10.1091/mbc.e10-05-0449 20943951 PMC2993750

[B15] Huang Y. Thoms J. A. Tursky M. L. Knezevic K. Beck D. Chandrakanthan V. (2016). MAPK/ERK2 phosphorylates ERG at serine 283 in leukemic cells and promotes stem cell signatures and cell proliferation. *Leukemia* 30 1552–1561. 10.1038/leu.2016.55 27055868 PMC5894814

[B16] Juliusson G. Hough R. (2016). Leukemia. *Prog. Tumor Res.* 43 87–100. 27595359 10.1159/000447076

[B17] Kang X. Qi Y. Zuo Y. Wang Q. Zou Y. Schwartz R. J. (2010). SUMO-specific protease 2 is essential for suppression of polycomb group protein-mediated gene silencing during embryonic development. *Mol. Cell* 38 191–201. 10.1016/j.molcel.2010.03.005 20417598 PMC2879644

[B18] Knudsen K. J. Rehn M. Hasemann M. S. Rapin N. Bagger F. O. Ohlsson E. (2015). ERG promotes the maintenance of hematopoietic stem cells by restricting their differentiation. *Genes Dev.* 29 1915–1929. 10.1101/gad.268409.115 26385962 PMC4579349

[B19] Kong X. T. Ida K. Ichikawa H. Shimizu K. Ohki M. Maseki N. (1997). Consistent detection of TLS/FUS-ERG chimeric transcripts in acute myeloid leukemia with t(16;21)(p11;q22) and identification of a novel transcript. *Blood* 90 1192–1199. 9242552

[B20] Kunz K. Wagner K. Mendler L. Holper S. Dehne N. Muller S. (2016). SUMO signaling by hypoxic inactivation of SUMO-specific isopeptidases. *Cell Rep.* 16 3075–3086. 10.1016/j.celrep.2016.08.031 27626674

[B21] Lima C. D. Reverter D. (2008). Structure of the human SENP7 catalytic domain and poly-SUMO deconjugation activities for SENP6 and SENP7. *J. Biol. Chem.* 283 32045–32055. 10.1074/jbc.m805655200 18799455 PMC2581585

[B22] Martelli M. P. Gionfriddo I. Mezzasoma F. Milano F. Pierangeli S. Mulas F. (2015). Arsenic trioxide and all-trans retinoic acid target NPM1 mutant oncoprotein levels and induce apoptosis in NPM1-mutated AML cells. *Blood* 125 3455–3465. 10.1182/blood-2014-11-611459 25795919

[B23] Martens J. H. (2011). Acute myeloid leukemia: a central role for the ETS factor ERG. *Int. J. Biochem. Cell Biol.* 43 1413–1416. 10.1016/j.biocel.2011.05.014 21664289

[B24] Moore S. D. Offor O. Ferry J. A. Amrein P. C. Morton C. C. Dal Cin P. (2006). ELF4 is fused to ERG in a case of acute myeloid leukemia with a t(X;21)(q25-26;q22). *Leuk. Res.* 30 1037–1042. 10.1016/j.leukres.2005.10.014 16303180

[B25] Ng A. P. Loughran S. J. Metcalf D. Hyland C. D. De Graaf C. A. Hu Y. (2011). Erg is required for self-renewal of hematopoietic stem cells during stress hematopoiesis in mice. *Blood* 118 2454–2461. 10.1182/blood-2011-03-344739 21673349

[B26] Qi Y. Wang J. Bomben V. C. Li D. P. Chen S. R. Sun H. (2014a). Hyper-SUMOylation of the Kv7 potassium channel diminishes the M-current leading to seizures and sudden death. *Neuron* 83 1159–1171. 10.1016/j.neuron.2014.07.042 25189211 PMC4877174

[B27] Qi Y. Zuo Y. Yeh E. T. Cheng J. (2014b). An essential role of small ubiquitin-like modifier (SUMO)-specific protease 2 in myostatin expression and myogenesis. *J. Biol. Chem.* 289 3288–3293. 10.1074/jbc.m113.518282 24344126 PMC3916531

[B28] Salek-Ardakani S. Smooha G. De Boer J. Sebire N. J. Morrow M. Rainis L. (2009). ERG is a megakaryocytic oncogene. *Cancer Res.* 69 4665–4673. 10.1158/0008-5472.can-09-0075 19487285

[B29] Singh A. A. Mandoli A. Prange K. H. Laakso M. Martens J. H. (2017). AML associated oncofusion proteins PML-RARA, AML1-ETO and CBFB-MYH11 target RUNX/ETS-factor binding sites to modulate H3ac levels and drive leukemogenesis. *Oncotarget* 8 12855–12865. 10.18632/oncotarget.14150 28030795 PMC5355061

[B30] Sotoca A. M. Prange K. H. Reijnders B. Mandoli A. Nguyen L. N. Stunnenberg H. G. (2016). The oncofusion protein FUS-ERG targets key hematopoietic regulators and modulates the all-trans retinoic acid signaling pathway in t(16;21) acute myeloid leukemia. *Oncogene* 35 1965–1976. 10.1038/onc.2015.261 26148230 PMC4833872

[B31] Stankiewicz M. J. Crispino J. D. (2013). AKT collaborates with ERG and Gata1s to dysregulate megakaryopoiesis and promote AMKL. *Leukemia* 27 1339–1347. 10.1038/leu.2013.33 23380710 PMC3915509

[B32] Thoms J. A. Birger Y. Foster S. Knezevic K. Kirschenbaum Y. Chandrakanthan V. (2011). ERG promotes T-acute lymphoblastic leukemia and is transcriptionally regulated in leukemic cells by a stem cell enhancer. *Blood* 117 7079–7089. 10.1182/blood-2010-12-317990 21536859

[B33] Tomita A. Kiyoi H. Naoe T. (2013). Mechanisms of action and resistance to all-trans retinoic acid (ATRA) and arsenic trioxide (As_2_O_3_) in acute promyelocytic leukemia. *Int. J. Hematol.* 97 717–725. 10.1007/s12185-013-1354-4 23670176

[B34] Tsuzuki S. Taguchi O. Seto M. (2011). Promotion and maintenance of leukemia by ERG. *Blood* 117 3858–3868. 10.1182/blood-2010-11-320515 21321361

[B35] Tursky M. L. Beck D. Thoms J. A. Huang Y. Kumari A. Unnikrishnan A. (2015). Overexpression of ERG in cord blood progenitors promotes expansion and recapitulates molecular signatures of high ERG leukemias. *Leukemia* 29 819–827. 10.1038/leu.2014.299 25306899

[B36] Varejao N. Lascorz J. Li Y. Reverter D. (2020). Molecular mechanisms in SUMO conjugation. *Biochem. Soc. Trans.* 48 123–135. 10.1042/bst20190357 31872228

[B37] Voisset E. Moravcsik E. Stratford E. W. Jaye A. Palgrave C. J. Hills R. K. (2018). Pml nuclear body disruption cooperates in APL pathogenesis and impairs DNA damage repair pathways in mice. *Blood* 131 636–648. 10.1182/blood-2017-07-794784 29191918 PMC5805489

[B38] Wu H. Chen X. Cheng J. Qi Y. (2016). SUMOylation and potassium channels: links to epilepsy and sudden death. *Adv. Protein Chem. Struct. Biol.* 103 295–321. 26920693 10.1016/bs.apcsb.2015.11.009

[B39] Yeh E. T. (2009). SUMOylation and De-SUMOylation: wrestling with life’s processes. *J. Biol. Chem.* 284 8223–8227. 10.1074/jbc.r800050200 19008217 PMC2659178

