# Supplementary material for: Hyper-SUMOylation of ERG Is Essential for the Progression of Acute Myeloid Leukemia
Source: Front Mol Biosci. 2021 Mar 26;8:652284. doi: 10.3389/fmolb.2021.652284 (PMC8032903; doi:10.3389/fmolb.2021.652284)
Supplement: Supplementary file 1 [file Data_Sheet_1.docx]

**Supplemental Tables**

**Table S1. Primers for plasmid construction**

| Plasmid | Sequence |
| --- | --- |
| ERG-K37R F | ACGCCACACCTGGCTAGAACAGAGATGACC |
| ERG-K37R R | CTTAGCCAGGTGTGGCGTTCCGTAGGCACA |
| ERG-K74R F | AGCCAGGGTCACCATCAGAATGGAATGTAA |
| ERG-K74R R | CTGATGGTGACCCTGGCTGGGGGTTGAGAC |
| ERG-K289R F | TCCTTCCACAGTGCCCAGAACTGAAGACCA |
| ERG-K289R R | CTGGGCACTGTGGAAGGAGATGGTTGAGCA |
| Flag-ERG F | CCAAGCTTATGATTCAGACTGTCCCGGACC |
| Flag-ERG R | CCCCCGGGTTAGTAGTAAGTGCCCAGATGA |
| pCDH-ERG F | ATTTGCGGCCGCATGATTCAGACTGTCCCGGACC |
| pCDH-ERG R | TGCTCTAGATTAGTAGTAAGTGCCCAGATGA |
| sh-ERG F | GATCCGGGAAGGAACTGTGCAAGACTTCCTGTCAG  ATCTTGCACAGTTCCTTCCCTTTTTG |
| sh-ERG R | AATTCAAAAAGGGAAGGAACTGTGCAAGATCTGACAGGAAGTCTTGCACAGTTCCTTCCCG |

**Table S2. Recombinant DNA**

| Plasmid | Source | Identifier |
| --- | --- | --- |
| 3XFLAG | Sigma-Aldrich | E4026 |
| FLAG-ERG | This paper | N/A |
| FLAG-ERG K37R | This paper | N/A |
| FLAG-ERG K74R | This paper | N/A |
| FLAG-ERG K289R | This paper | N/A |
| FLAG-ERG K37R&K74R&K289R | This paper | N/A |
| FLAG-SENP1 | This paper | N/A |
| HA-PIAS1 | This paper | N/A |
| HA-PIAS2α | This paper | N/A |
| HA-PIAS2β | This paper | N/A |
| HA-PIAS3 | This paper | N/A |
| HA-PIAS4 | This paper | N/A |
| HA-SUMO1 | Addgene | 17359 |
| HA-SUMO2 | This paper | N/A |
| HA-Ubiquitin | This paper | N/A |
| p-GREEN-PURO | [System Biosciences](http://www.integratedsci.com.au/brands/system-biosciences.html) | MZIP000-PA-1 |
| pCDH-ERG | This paper | N/A |
| pCDH-ERG K37R&K74R& K289R | This paper | N/A |
| RGS-SENP2w | This paper | N/A |
| RGS-SENP2m | This paper | N/A |
| RGS-SENP3 | This paper | N/A |
| sh-ERG | This paper | N/A |

**Table S3. Primers for quantitative real-time PCR**

| Primer | Sequence |
| --- | --- |
| β-Actin F | GAGGTATCCTGACCCTGAAGTA |
| β-Actin R | CACACGCAGCTCATTGTAGA |
| Ccnb1 F | TCTTCTCGAATCGGGGAAC |
| Ccnb1 R | GACCTTGGCCTTATTTTCTGC |
| Cks2 F | TCGATGAGCACTACGAGTACC |
| Cks2 R | CCATCCTAGACTCTGTTGGACAC |
| Dkc1 F | AAAGACCGGAAGCCATTACAAG |
| Dkc1 R | GCCACTGAGAAGTGTCTAATTGA |
| ERG F | ACACCGTTGGGATGAACTAC |
| ERG R | GATCTGCTGGCACGATAACT |
| Myc F | GCGACTCTGAAGAAGAGCAAGA |
| Myc R | GACCTCTTGGCAGGGGTTTG |
| Pigp F | ATGGTGGAAAATTCACCGTCG |
| Pigp R | ACGAAAGCCCACACAAGATAAA |
| Pold2 F | ACCTGTGGCAACTTACACCAA |
| Pold2 R | GTGGCATAAATATGGGCGTACT |
| Tfrc F | GTGAAACTGGCTGAAACGGAG |
| Tfrc R | GGTCTGCCCAATATAAGCGAGA |

**Table S4. Antibodies for Western blotting and co-IP assay**

| Name | Identifier | Source | Species |
| --- | --- | --- | --- |
| β-Actin | AB0011 | Abways | Mouse |
| β-Tubulin | 10094-1-AP | Proteintech | Mouse |
| CD11b | 550019 | BD pharmigen | Mouse |
| ERG | 97249S | Cell Signaling | Rabbit |
| FLAG | F1804 | Sigma-Aldrich | Mouse |
| HA | 3724 | Cell Signaling | Rabbit |
| PML | 33156S | Cell Signaling | Rabbit |
| SENP2 | sc-67075 | Santa Cruz | Rabbit |
| SENP3 | 5591S | Cell Signaling | Rabbit |
| SUMO2/3 | ab81371 | Abcam | Mouse |
| Ubiquitin | 3933 | Cell Signaling | Rabbit |
